# Hydrophobic Silica Aerogel with Higher Flame Retardancy, Thermal Radiation Shielding, and High-Temperature Insulation Properties Through Introduction of TiO_2_

**DOI:** 10.3390/gels11040249

**Published:** 2025-03-27

**Authors:** Huiying Sun, Yuelei Pan, Song He, Lunlun Gong, Zhongxin Zhang, Xudong Cheng, Heping Zhang

**Affiliations:** 1 State Key Laboratory of Fire Science, University of Science and Technology of China, Hefei 230027, China; sunhuiying@mail.ustc.edu.cn (H.S.); gongll@ustc.edu.cn (L.G.); zhxinzh@mail.ustc.edu.cn (Z.Z.); chengxd@ustc.edu.cn (X.C.); zhanghp@ustc.edu.cn (H.Z.); 2 School of Resources and Environmental Engineering, Wuhan University of Technology, Wuhan 430070, China

**Keywords:** hydrophobic silica aerogel, flame retardancy, titanium dioxide, thermal radiation, combustion characteristics

## Abstract

SiO_2_ aerogels have garnered significant attention for thermal insulation applications due to their exceptional hydrophobicity and thermal resistance. However, the organic functional groups enabling hydrophobicity introduce flammability concerns, limiting their safe implementation in high-temperature environments. This study presents a novel TiO_2_ doping strategy (SA/TiO_2_) that simultaneously enhances thermal safety while preserving the material’s intrinsic advantages. The optimized SA/TiO_2_ composite demonstrates remarkable fire resistance, achieving a 44% reduction in gross calorific value (GCV) and a 25.4% decrease in total heat release (THR) compared to conventional aerogels. Thermogravimetric analysis reveals substantial thermal stability improvements, with TiO_2_ incorporation elevating the initial and peak decomposition temperatures by 207 °C and 167 °C, respectively. When integrated into fiber-reinforced SiO_2_ aerogel composites, the 10% TiO_2_-doped formulation achieves an ultra-low GCV of 2.75 MJ/kg while maintaining superior insulation performance (~18 mW/m·K). Notably, the composite demonstrates exceptional high-temperature stability, retaining minimal thermal conductivity of 25.5 mW/m·K at 600 °C. The titanium dioxide phase effectively attenuates thermal radiation transmission while preserving the matrix’s nanoporous architecture, thereby synergistically enhancing both fire safety and thermal insulation capabilities in demanding operational environments.

## 1. Introduction

Silica aerogel, an emerging nanomaterial characterized by its lightweight nature, porous architecture, and three-dimensional network structure, has garnered significant attention across multiple industries. With exceptional properties including ultralow thermal conductivity (0.017–0.021 W/m·K), remarkably low density (0.03–0.50 g/cm^3^), and high specific surface area (500–1200 m^2^/g) [1,2,3,4], this advanced material demonstrates versatile applications spanning aerospace engineering, building insulation systems, petrochemical operations, and renewable energy technologies [5,6,7]. The unique combination of these physical characteristics positions silica aerogel as a promising solution for next-generation thermal management challenges in both industrial and commercial sectors.

In ambient pressure drying processes for silica aerogel preparation, surface modification with hydrophobic organic groups plays a crucial role in minimizing capillary tension during drying, thereby effectively reducing skeleton collapse-induced volume shrinkage [1,8,9]. This surface functionalization concurrently mitigates surface hydroxyl reactivity, preventing structural degradation caused by moisture absorption during operational use and ensuring optimal thermal insulation performance in practical applications [10,11]. However, when exposed to thermal radiation (200–500 °C) under aerobic conditions, the organic constituents (-CH_3_, -C_2_H_5_) in these aerogels undergo thermal oxidation, generating significant quantities of combustible volatile compounds [12,13]. These volatiles accumulate until reaching critical concentrations where ambient oxygen and temperatures exceeding ignition thresholds trigger combustion. Subsequent heat feedback from combustion zones accelerates thermal oxidation rates and fuel fragment generation, creating a self-sustaining cycle that ultimately leads to complete aerogel consumption [14]. The fundamental fire risk of these materials stems from this high-temperature pyrolysis mechanism and subsequent volatile release. While aerogel composites are classified as Class A refractory materials (GCV ≤ 3 MJ/kg), commercial silica aerogel mats frequently fail to meet this standard. Early investigations by Redouane et al. [15] systematically characterized the thermal hazards of silica aerogels through comprehensive analysis of pyrolysis behavior, flammability profiles, and oxidation kinetics. These findings underscore that high-temperature combustion and pyrolysis remain persistent challenges limiting the safe application of silica aerogel materials.

In recent years, strategies to mitigate the flammability of silica aerogels have focused on regulating organic group content within their framework and incorporating flame-retardants such as metal hydroxides and nitrogen–phosphorus compounds. Critical progress has been achieved through suppressing pyrolysis and combustion of organic components at elevated temperatures, as demonstrated in studies addressing thermal degradation mechanisms [16,17,18,19]. A notable advancement involves substituting trimethylchlorosilane with dimethyldichlorosilane as a surface modifier, which significantly decreases combustible surface groups and lowers ignition risks [20]. The inorganics, Al(OH)_3_ and Mg(OH)_2_, have been used as physical dopants to reduce the flammability of hydrophobic silica aerogels. The effectiveness of Al(OH)_3_ and Mg(OH)_2_ for reducing the heat release rate for combustion has been demonstrated. The combustion inhibition effect of Mg(OH)_2_ is better than that of Al(OH)_3_ [21,22,23]. The thermal stability of transparent silica aerogels has been improved by using Al_2_O_3_ atomic layer deposition to provide a face modification [24]. A novel nitrogen–phosphorus flame-retardant, DOPO-vts, has been introduced to the surface of a silica aerogel by co-hydrolysis condensation [25]. The formation of P-O-Si bonds endows aerogels with cooperative flame retardancy. Aerogels containing these entities exhibit excellent flame retardancy and enhanced thermal stability. Nevertheless, these enhancement strategies—whether involving modifier substitution or flame-retardant integration—present an inherent trade-off: while effectively improving thermal stability, they concurrently increase material density and thermal conductivity, ultimately compromising the fundamental thermal insulation performance of hydrophobic SiO_2_ aerogels.

TiO_2_-doped hydrophobic silica aerogel (HSA) powders and blend blankets (HSABs) were successfully synthesized through a classical sol–gel process using both organic and inorganic silicon sources. Comprehensive characterization of these materials included microstructural analysis, pore structure evaluation, and assessments of thermal insulation properties, thermal stability, and fire safety. Systematic investigation revealed that SA/TiO_2_ aerogel composites with 10% TiO_2_ doping demonstrated exceptional performance: achieving a remarkably low gross calorific value (GCV) of 2.75 MJ/kg, ultralow thermal conductivity of 0.025 W/(m·K) at 600 °C, and superior room-temperature insulation capability (1.8 W/(m·K) at 60 °C) surpassing air’s thermal conductivity. This study conclusively demonstrates TiO_2_’s dual functionality in simultaneously reducing the calorific value of silica aerogels while enhancing their thermal stability and safety parameters. The findings substantiate the strategic incorporation of TiO_2_ as an effective modifier for optimizing aerogel thermal management properties, thereby advancing practical applications of hydrophobic silica aerogels in high-performance thermal insulation systems.

## 2. Results and Discussion

### 2.1. Microstructure and Pore Structure

**Figure 1:**
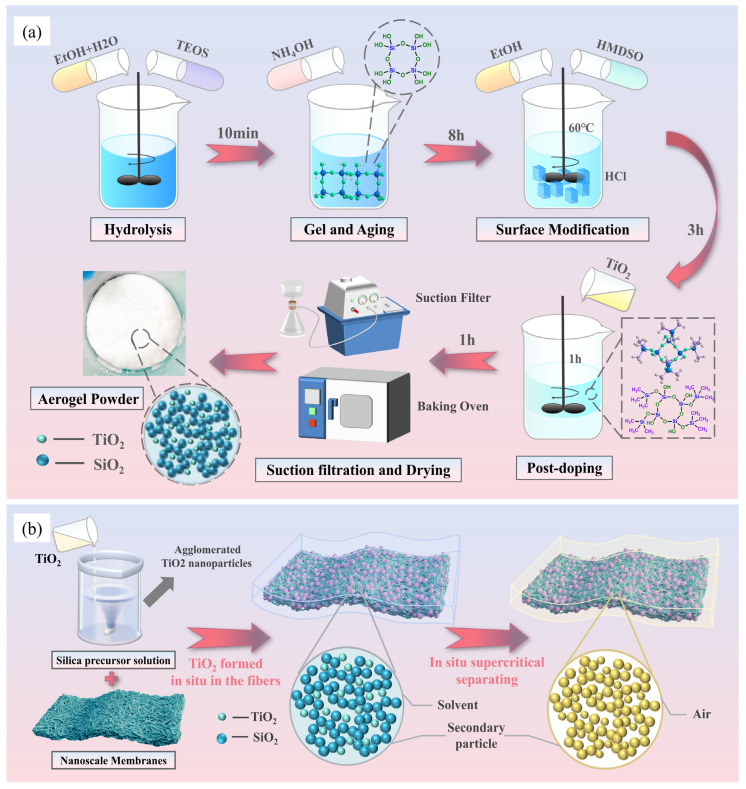
The schematic diagram of the preparation of (**a**) SA/TiO_2_ aerogel powder and (**b**) SA/TiO_2_ aerogel blanket.

Hydrophobic SA/TiO_2_ aerogel powders and composite materials were successfully synthesized via a tetraethyl orthosilicate (TEOS)-based precursor system under ambient pressure. Figure 1a illustrates the preparation process of TiO_2_-doped SiO_2_ aerogel powder, where TiO_2_ nanoparticles were incorporated during the sol–gel phase. Subsequent solvent exchange, surface hydrophobization, and ambient-pressure drying yielded low-thermal-conductivity aerogel powders with reduced calorific values. Notably, Figure 1b presents a comparative study using TEOS and sodium silicate (water glass) as alternative silica precursors to demonstrate the universal applicability of TiO_2_ opacifiers in suppressing the gross calorific value (GCV) of silica aerogel composites while simultaneously investigating precursor effects on combustion performance. The synthesized powders were systematically labeled as SA/TiO_2_-1 to SA/TiO_2_-4 based on their TiO_2_ mass fractions (5%, 10%, 15%, 20%). Parallel composite systems, designated SA/TiO_2_(A) and SA/TiO_2_(B) for TEOS-derived and water glass-derived materials, respectively, were formulated with incremental TiO_2_ loadings (2.5%, 5%, 7.5%, 10%). These specimens follow a standardized nomenclature: SA/TiO_2_(A)-1 through SA/TiO_2_(A)-4 and SA/TiO_2_(B)-1 through SA/TiO_2_(B)-4, corresponding to ascending TiO_2_ concentrations.

**Figure 2:**
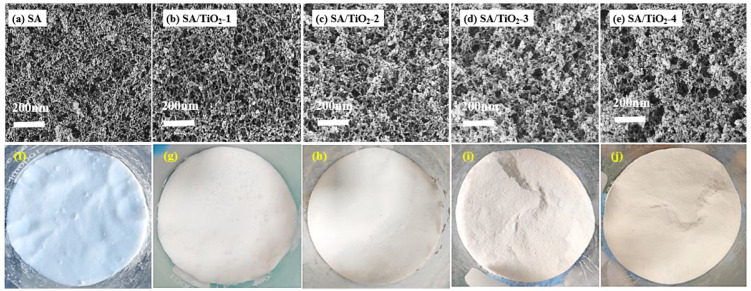
Microstructures of (**a**,**f**) SA, (**b**,**g**) SA/TiO_2_-1, (**c**,**h**) SA/TiO_2_-2, (**d**,**i**) SA/TiO_2_-3, and (**e**,**j**) SA/TiO_2_-4.

Figure 2a–e present the microstructural evolution of pure SA and TiO_2_-incorporated SA/TiO_2_ aerogels, with corresponding macroscopic features displayed in Figure 2f–j. The pure SA matrix (Figure 2a) demonstrates a characteristic silica network architecture comprising dense amorphous silica primary particles (1–2 nm) formed through silicon source polycondensation. These nanoparticles aggregate into spherical secondary particles (5–10 nm) that interconnect to establish a three-dimensional pearl necklace-like framework [26,27]. Comparative analysis of Figure 2b–e reveals that all TiO_2_-doped variants maintain the fundamental nanoporous network structure observed in pure SA. Notably, increasing TiO_2_ loading induces particle agglomeration within the aerogel matrix, accompanied by progressive broadening of pore size distribution and enhanced specific surface area—phenomena attributed to interfacial interactions between TiO_2_ nanoparticles [28]. Macroscopically, Figure 2f–j document a systematic color transition from transparent pale blue to opaque pale yellow with rising TiO_2_ content, while maintaining uniform powder morphology. This chromatic evolution, proportional to dopant concentration, occurs without significant microstructural alteration, confirming that TiO_2_ incorporation minimally impacts the material’s fundamental architecture.

**Figure 3:**
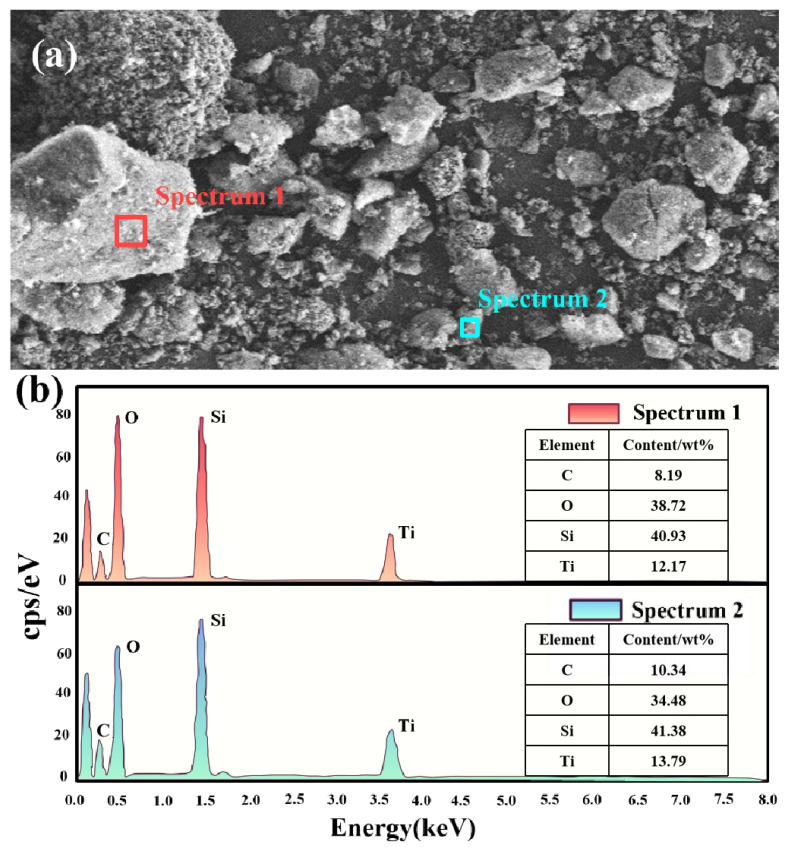
(**a**) The representative SEM micrograph and (**b**) the corresponding EDS spectrum of the 20 wt% SA/TiO_2_ composite.

To elucidate the composite’s microstructure, SA/TiO_2_-4 was analyzed via SEM and EDS. Figure 3a depicts the SEM image of SA/TiO_2_-4, with two representative regions selected for elemental analysis: a large consolidated block and a smaller fragmented area. EDS results (Figure 3b) demonstrate identical primary elements (C, O, Ti, Si) in both regions, with comparable Ti concentrations across the sampled areas. The observed peak at ~2.1 eV corresponds to Pt, originating from sputter-coating during SEM sample preparation. These findings validate the successful integration and homogeneous dispersion of TiO_2_ within the silica aerogel matrix, confirming the structural integrity of the composite powders.

**Figure 4:**
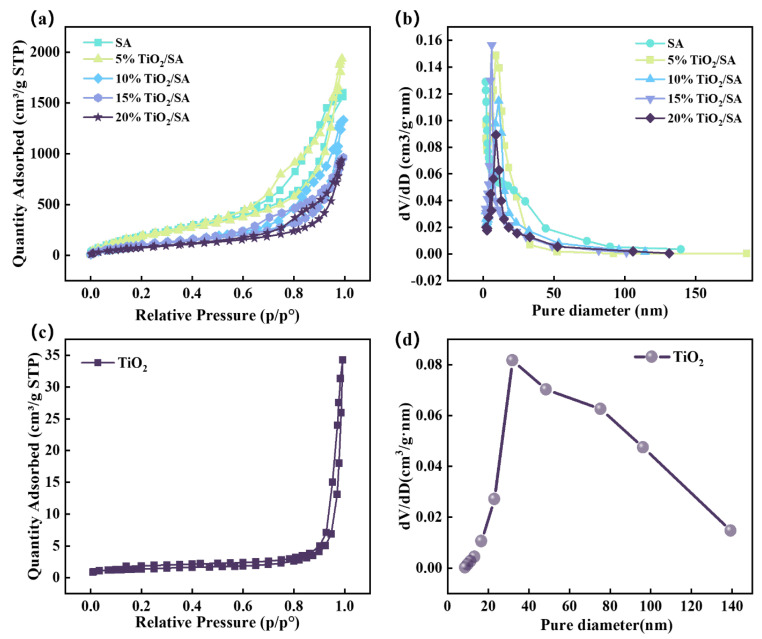
N_2_ adsorption isotherms (**a**) and pore size distribution (**b**) of SA and SA / TiO_2_, and N_2_ adsorption isotherms (**c**) and pore size distribution (**d**) of TiO_2_.

Figure 4 presents the nitrogen adsorption–desorption isotherms and pore structure characteristics of TiO_2_, SA, and SA/TiO_2_ samples. All specimens display type IV isotherms with H3-type hysteresis loops under IUPAC classification [29], characteristic of mesoporous materials (2–50 nm pore range) [30]. As demonstrated in Figure 4a,b, the nitrogen adsorption capacity and pore volume of SA/TiO_2_ composites exhibit a progressive decline with increasing TiO_2_ doping levels, reinforcing that TiO_2_ incorporation alters aerogel pore architecture. This modification manifests as reduced specific surface area and expanded pore dimensions. Comparative analysis of Figure 4b–d reveals distinct pore size distributions: TiO_2_ displays dominant pores near 40 nm, whereas pure SA and SA/TiO_2_ exhibit primary distributions around 10 nm. The SA/TiO_2_ composites show narrower and more pronounced peaks in their pore size distribution profiles, indicative of enhanced uniformity and spatial confinement of the aerogel pore structure compared to pristine components.

The BET surface area, pore volume, and average pore size data are summarized in Table 1. Notably, increasing TiO_2_ content correlates with a progressive decline in the specific surface area of SA/TiO_2_ composites, while both pore volume and average pore size exhibit a concurrent upward trend. This behavior corroborates that TiO_2_ incorporation disrupts silica skeleton formation [23,31], as evidenced by structural collapse within the aerogel network during ambient-pressure drying. Such morphological reorganization facilitates the development of internal macropores, consistent with the observed inverse relationship between surface area and pore dimensions.

### 2.2. Basic Physicochemical Properties

**Figure 5:**
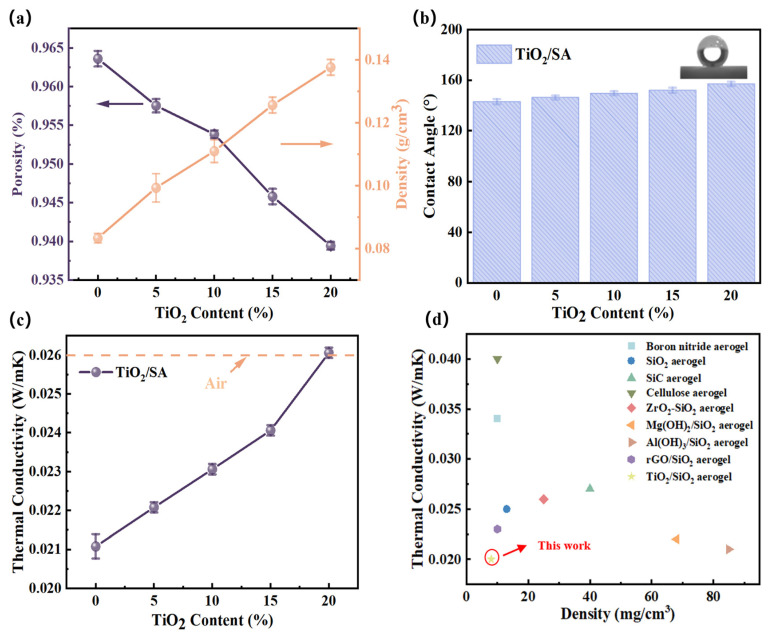
(**a**) Density and porosity characteristics, (**b**) contact angle measurements, and (**c**) thermal conductivity performance of SA/TiO_2_ composite powders as functions of TiO_2_ content, along with (**d**) comparative thermal conductivity versus density relationships for various aerogel-like materials.

Figure 5 systematically illustrates the variations in bulk density, porosity, water contact angle, and thermal conductivity of SA/TiO_2_ composites as a function of TiO_2_ loading. As depicted in Figure 5a, the bulk density of SA/TiO_2_ exhibits a pronounced increase (75% enhancement to 1.4 g/cm^3^) when the TiO_2_ content rises from 5% to 20%, attributable to the inherently higher density of TiO_2_ compared to pristine silica aerogels. Concurrently, the porosity demonstrates an inverse trend, decreasing from 96.1% to 93.99%, a result consistent with partial structural collapse of the aerogel framework due to TiO_2_ incorporation. Hydrophobicity, quantified via water contact angle measurements (Figure 5b), reveals that pure SA maintains a contact angle exceeding 145°, while SA/TiO_2_ composites display further hydrophobicity enhancement with increasing TiO_2_ content. Thermal conductivity, a critical parameter for insulation performance (Figure 5c), proportionally rises with TiO_2_ loading. This phenomenon arises from TiO_2_ particles creating additional heat transfer pathways [32,33], despite their capacity to mitigate radiative heat loss. Notably, SA/TiO_2_ composites with TiO_2_ concentrations below 15% retain thermal conductivities below that of static air (~26 mW/m·K at ambient conditions). These findings underscore the feasibility of tailoring TiO_2_ content to optimize insulation properties while balancing structural integrity and thermal performance.

To demonstrate the superior thermal insulation properties of SA/TiO_2_ composites, Figure 5d comparatively presents thermal conductivity–density correlations of state-of-the-art aerogel materials reported in the literature [18,19,34,35]. The novel SA/TiO_2_ composite containing 10 wt% SA exhibits a lower density (0.11 g/cm^3^) and exceptionally reduced thermal conductivity (23 mW/(m·K)), outperforming all referenced aerogel systems. This remarkable dual-parameter advantage positions SA/TiO_2_ composites as highly promising candidates for advanced lightweight thermal management applications, particularly where simultaneous demands for mass minimization and thermal insulation optimization must be satisfied [36].

**Figure 6:**
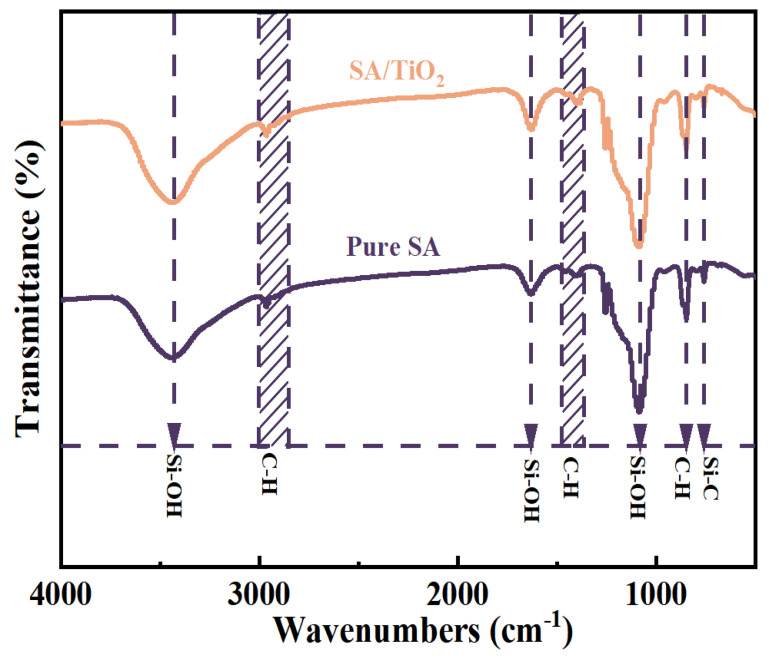
The FTIR of pure SA and 20% TiO_2_ /SA.

Figure 6 displays the FTIR spectra of pure SA and SA/TiO_2_-4. Characteristic absorption bands at 3450 cm^−1^ and 1630 cm^−1^ are assigned to Si-OH groups and adsorbed water [37], while the peak near 2970 cm^−1^ corresponds to asymmetric and symmetric C-H bond stretching [10,38]. The presence of a Si-C bond at 758 cm^−1^ confirms methyl group attachment on the SA framework [29]. Crucially, SA/TiO_2_ and pure SA share nearly identical spectral profiles aside from intensity variations, with no emergent chemical bonds detected. These observations confirm that the SA-TiO_2_ interaction is limited to physical blending rather than chemical bonding [39].

**Figure 7:**
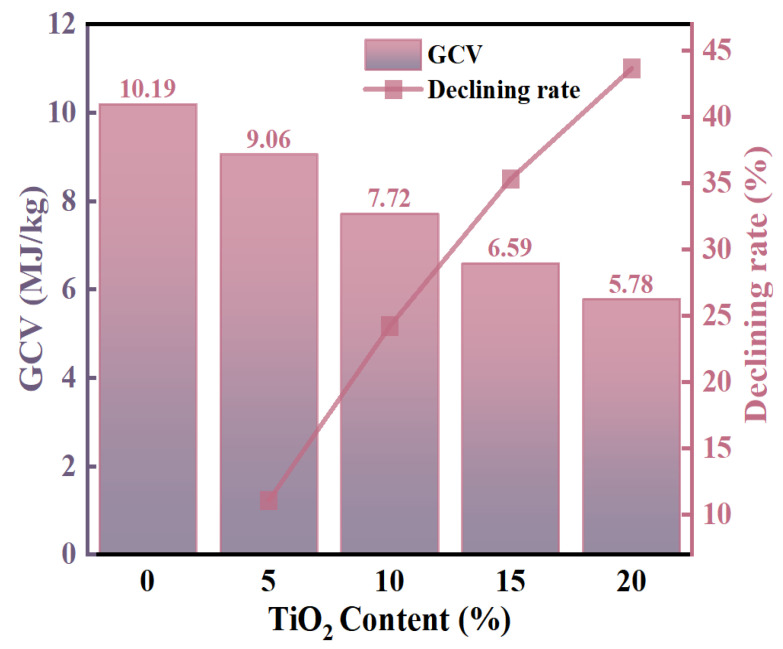
GCV of the pure SA and SA/TiO_2_ powders along with the TiO_2_ content.

The gross calorific value (GCV), defined as the total heat energy released through complete combustion of a unit mass of fuel [40,41], serves as a critical thermochemical parameter for evaluating fuel energy content. As demonstrated in Figure 7, the GCV of SA/TiO_2_ composites exhibits a pronounced negative correlation with TiO_2_ content. Notably, at 20% TiO_2_ incorporation, the composite’s GCV decreases to approximately 5.78 MJ/kg—representing a 44% reduction compared to pure SA (10.19 MJ/kg). This significant GCV attenuation suggests a corresponding reduction in thermal hazard risks during combustion processes, indicating that SA/TiO_2_ composites demonstrate enhanced safety characteristics relative to the pure SA matrix.

### 2.3. Conbustion Behaviors

#### 2.3.1. Thermal Analysis

**Figure 8:**
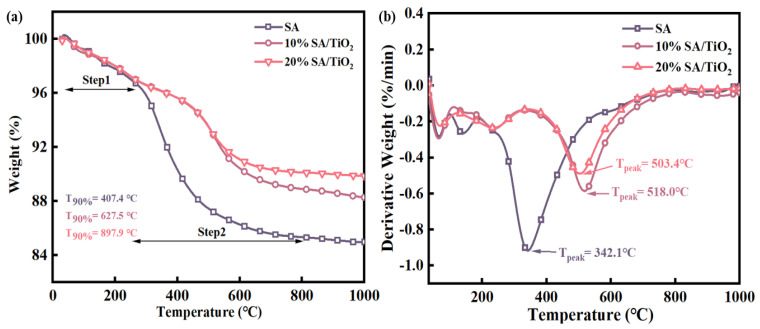
TG curve (**a**) and DTG curve (**b**) of SA and SA / TiO _2_ composite powders.

The thermogravimetric (TG) analysis method was employed to evaluate the thermal stability of samples, which reveals physical changes and chemical reactions during heating/cooling processes through continuous mass–temperature relationship monitoring. Figure 8 presents the TG profiles of pure SA and SA/TiO_2_ composites (Samples 1–4) under air atmosphere at a 20 °C/min heating rate. Both pure SA and SA/TiO_2_ exhibit negligible mass loss (100–150 °C) attributed to evaporation of residual water and organic solvents [42,43]. As shown in Figure 8a, pure SA demonstrates significant mass loss accompanied by distinct exothermic peaks above 340 °C, corresponding to thermal oxidative decomposition of the organic modifier (-Si-CH_3_) on the SA framework [2,44]. While 10% SA/TiO_2_ and 20% SA/TiO_2_ follow similar decomposition trends to pure SA with single-stage mass loss from ambient to 1000 °C and corresponding DTG exothermic peaks, notable improvements are observed in their thermal decomposition parameters. The initial decomposition temperature (Tonset) and peak decomposition temperature (Tpeak) progressively increase with TiO_2_ content: When TiO_2_ loading rises from 5% to 20%, Tonset elevates from 264 °C to 471 °C (*Δ* + 207 °C) and Tpeak shifts from 342 °C to 518 °C (*Δ* + 176 °C), representing approximately 200 °C enhancement compared to pure SA. This significant temperature delay in both Tonset and Tpeak confirms that TiO_2_ doping effectively enhances the thermal stability of the SA matrix while preserving its hydrophobic properties. Importantly, the absence of additional decomposition events during pyrolysis confirms the structural integrity of TiO_2_ components. These thermal stability improvements substantially reduce the potential hazards of SA/TiO_2_ composites compared to pure SA, making them safer for high-temperature applications.

#### 2.3.2. Flame-Retardant Properties

Microscale combustion calorimetry (MCC) represents a sophisticated analytical approach for quantitatively evaluating the flame-retardant performance of finite-sized aerogels under controlled combustion conditions. This standardized methodology enables direct measurement of critical combustion parameters, including total heat release and essential flammability indices, while simultaneously observing gas-phase and condensed-phase combustion behavior within a pyrolysis–combustion flow reactor [45]. Experimental conditions comprised a temperature ramp from ambient to 900 °C at a heating rate of 1 °C·s^−1^, with combustion sustained at 900 °C under an 80 mL·min^−1^ airflow. Key evaluation parameters encompass heat release rate (HRR), quantifying thermal energy generation during material decomposition; peak HRR (pHRR), indicating maximum combustion intensity; total heat release (THR), calculated through integration of HRR curves; and heat release capacity (HRC), defined as the HRR peak normalized by heating rate [46]. These thermochemical metrics collectively provide mechanistic insights into material flammability and energy release patterns.

**Figure 9:**
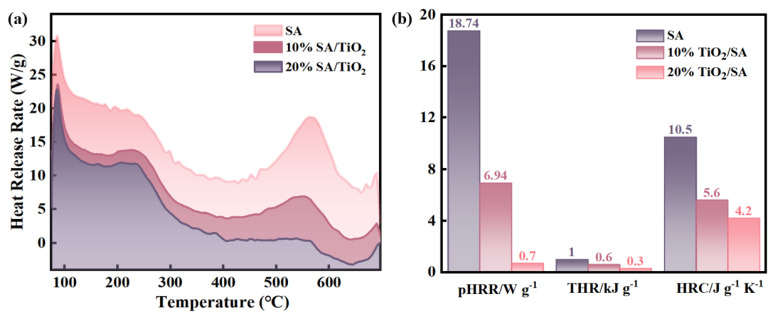
(**a**) MCC thermograms of pure SA and TiO_2_/SA composites (10% and 20% loadings), and (**b**) comparative flame retardancy parameters (pHRR, THR, HRC) across the materials.

Figure 9a presents the HRR profile as a function of pyrolysis temperature for the aerogel systems. The HRR curves demonstrate a progressive reduction in thermal output with increasing TiO_2_ doping concentration. Two distinct exothermic events are observed during pyrolysis: an initial heat release at approximately 200 °C corresponding to solvent/water evaporation, followed by a secondary exothermic peak between 500 and 600 °C for pure SA, 10% SA/TiO_2_, and 20% SA/TiO_2_ composites. This secondary thermal event exhibits a decreased slope that correlates with thermogravimetric (TG) analysis patterns. Notably, the second exothermic peak of SA aerogel is sharper, while the exothermic peaks of 10% SA/TiO_2_ and 20% SA/TiO_2_ are milder, which further indicates that the introduction of TiO_2_ reduces the thermal decomposition of aerogel. A final decomposition event emerges near 700 °C across all samples, consistent with residual mass loss patterns in TG curves. Quantitative analysis reveals significant flame retardancy improvements: peak HRR (pHRR) values dramatically decrease from 18.74 W/g (pure SA) to 6.94 W/g (10% composite) and 0.7 W/g (20% composite). The 20% SA/TiO_2_ formulation achieves a 46% reduction in total heat release (THR) and 96% suppression of pHRR compared to pristine SA. Concurrently, the heat release capacity (HRC) displays TiO_2_-concentration-dependent attenuation, confirming the enhanced flame inhibition efficacy. These collective findings establish SA/TiO_2_ aerogels as superior flame-retardant materials characterized by suppressed thermal decomposition dynamics, attenuated fire potential, and significantly reduced total heat generation during combustion.

#### 2.3.3. Composition of Pyrolysis Products

**Figure 10:**
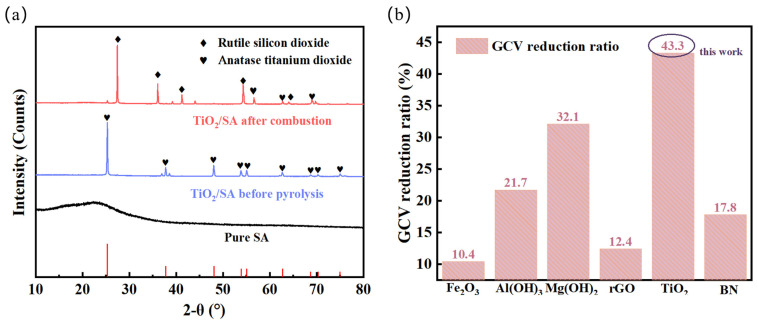
(**a**) XRD patterns of pure SA and its 20% TiO_2_-containing composite and (**b**) GCV reduction ratios of SA subjected to different treatment approaches.

Figure 10a presents the XRD patterns of SA, the SA/TiO_2_-4 composite, and its pyrolyzed counterpart. Notably, SA retains its amorphous structural integrity both pre- and post-pyrolysis, consistent with previous studies demonstrating SA’s resistance to crystallization even under 1200 °C thermal treatment [36,47]. The incorporation of TiO_2_ introduces distinct crystalline signatures before pyrolysis, aligning with Yang et al.’s report [31] that TiO_2_ exhibits oxidation stability up to 1300–1500 °C and decomposition resistance exceeding 1800 °C. It is worth noting that the crystal phase of anatase TiO_2_ changed obviously before and after pyrolysis, and most of them were transformed into rutile TiO_2_, which was mainly related to the crystal phase ratio of TiO_2_ at different pyrolysis temperatures [36]. Therefore, TiO_2_ can maintain its good crystal structure in the process of silicon aerogel pyrolysis without decomposition and has excellent high-temperature resistance and thermal stability. This can preliminarily infer the mechanism of titanium dioxide inhibiting the combustion of aerogels, that is, when the temperature rises to the pyrolysis temperature of silica aerogels, titanium dioxide can use its excellent shading performance to reduce the radiation heat transfer through aerogels. By cutting off the high-temperature heat transfer path, the combustion possibility of the aerogel is reduced, and the thermal stability and thermal safety of the hydrophobic silica aerogel are effectively improved.

Figure 10b comparatively evaluates gross calorific value (GCV) attenuation efficiencies of prevalent SA modifiers at equivalent 20 wt% loading levels. The SA/TiO_2_ composite demonstrates a remarkable 43.3% GCV reduction relative to pristine SA, substantially outperforming conventional physical additives. This pronounced calorific suppression directly evidences the composite’s superior flame-retardant efficacy, attributable to TiO_2_’s catalytic char-forming mechanism and synergistic smoke-suppression effects within the SA matrix [47].

### 2.4. SA/TiO_2_ Aerogel Composites

#### 2.4.1. Density and Hydrophobicity

**Figure 11:**
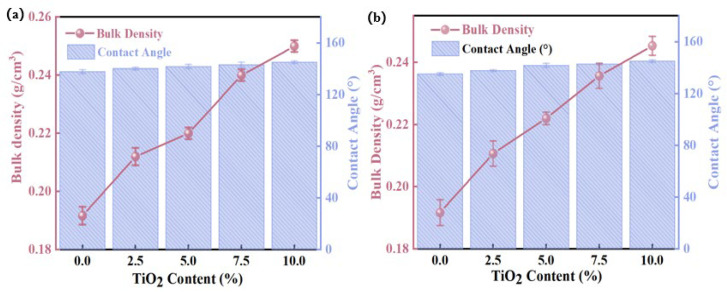
Variations in bulk density and contact angle of SA/TiO_2_ composites as functions of TiO_2_ content, fabricated with distinct silica precursors: (**a**) water glass and (**b**) tetraethyl orthosilicate (TEOS) as respective silicon sources.

As illustrated in Figure 11, SA/TiO_2_ composites exhibit monotonic increases in bulk density and hydrophobic contact angle with rising TiO_2_ doping levels, irrespective of the silicon source employed—a trend consistent with SA/TiO_2_ powder behavior. Notably, at 10% TiO_2_ loading, the composite density reaches 0.24 g/cm^3^, representing a mere 25% increase over pure SA while retaining ultralight characteristics. Concurrently, the composites demonstrate exceptional hydrophobicity (contact angle > 120°), a property directly attributable to TiO_2_’s intrinsic water-repellent nature and its synergistic interfacial interactions with the SA matrix.

#### 2.4.2. GCV and Thermal Insulation Performance

**Figure 12:**
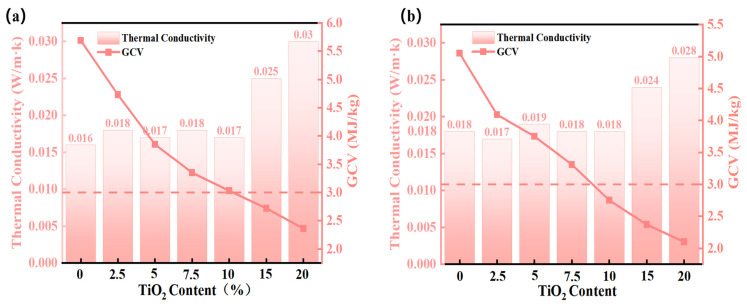
Thermal conductivity and GCV of pure SA and SA/TiO_2_ composites synthesized with (**a**) TEOS and (**b**) water glass as silicon sources.

Figure 12a,b present comparative analyses of thermal insulation performance and GCV for SA/TiO_2_ aerogel composites synthesized using water glass and TEOS as silicon sources. Notably, pure SA exhibits a GCV exceeding 5 MJ/kg regardless of the silicon source employed. Upon incorporation of 5% TiO_2_, SA/TiO_2_ (A) demonstrates a 32.3% GCV reduction, while SA/TiO_2_ (B) shows a 29.7% decrease. Further increasing TiO_2_ content to 10% amplifies this suppression effect, with GCV reductions of 46.7% (3.03 MJ/kg) and 45.5% (2.75 MJ/kg) for SA/TiO_2_ (A) and (B), respectively. Beyond this threshold, the composites exhibit a linear correlation between TiO_2_ loading and GCV diminution. These findings confirm TiO_2_’s significant role in mitigating the flammability of SA matrices, thereby substantially enhancing thermal safety. The superior GCV suppression observed in SA/TiO_2_ (B) compared to its counterpart (A) aligns with the intrinsic low-calorific characteristics of water glass-derived aerogels, underscoring silicon source selection as a critical design parameter for fire-safe composite optimization.

**Figure 13:**
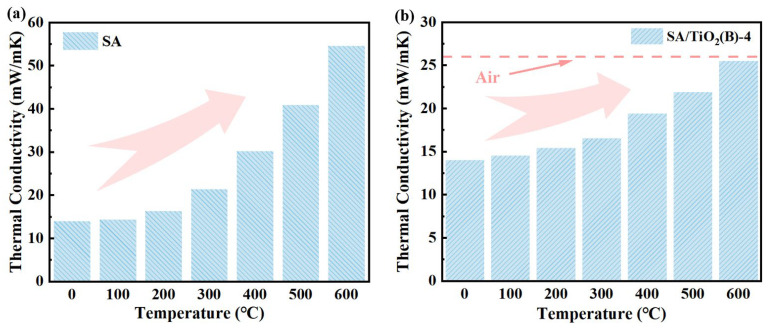
Temperature-dependent thermal conductivities of (**a**) pure SA and (**b**) SA/TiO_2_ composite (10 wt% TiO_2_).

Thermal conductivity serves as a critical parameter for evaluating the thermal insulation performance of aerogel composites. As illustrated in Figure 13a,b, all tested SA/TiO_2_ composites with TiO_2_ content below 10% maintained low thermal conductivity levels (<0.020 W/(m·K)), indicating minimal impact of TiO_2_ incorporation on the intrinsic insulation properties of the aerogel matrix. However, increasing TiO_2_ content to 15% and 20% triggered a substantial rise in thermal conductivity, with values reaching 0.030 W/(m·K) and 0.028 W/(m·K), respectively, at 20% doping. Notably, the 10% TiO_2_-doped SA/TiO_2_ (B) composite achieved dual functional superiority: it retained an ultralow thermal conductivity of 0.020 W/(m·K) while attaining a gross calorific value (GCV) of 2.75 MJ/kg. This performance meets the Class A incombustibility criteria specified in GB 8624-2012 [48], effectively resolving the fire safety limitations inherent in conventional silica aerogels and facilitating their broader application in high-performance thermal insulation systems.

To verify TiO_2_’s inhibitory effect on radiative heat transfer in aerogels at elevated temperatures, we systematically measured the thermal conductivity evolution from room temperature to 600 °C for four SA/TiO_2_ aerogel composites (10 wt% dopant) alongside pure SA. As illustrated in Figure 10, both pure SA and 10% SA/TiO_2_ exhibit temperature-dependent conductivity increases yet demonstrate fundamentally distinct behaviors. The pure SA system (Figure 10a) maintains exceptional insulation below 300 °C (<20 mW/m·K) but suffers catastrophic thermal degradation above 400 °C, culminating in 50+ mW/m·K thermal conductivity at 600 °C due to the structural collapse of its 3D network. This sharply contrasts with TiO_2_-modified composites (Figure 10b), where the 10% SA/TiO_2_ retains remarkably low thermal conductivity (25.5 mW/m·K at 600 °C)—outperforming even ambient air thermal resistance. The preserved structural integrity and suppressed radiation transport in TiO_2_-containing aerogels confirm their superior high-temperature insulation capabilities.

#### 2.4.3. Flame-Retardant Mechanism

Furthermore, the infrared radiation suppression capability of TiO_2_ opacifiers in the aerogel was quantitatively evaluated using infrared transmittance and the mass-specific extinction coefficient. A higher extinction coefficient directly correlates with enhanced infrared radiation attenuation and reduced radiative thermal conductivity. The mass-specific extinction coefficient was derived from the infrared transmittance measurements of the synthesized nanofiber aerogel, demonstrating TiO_2_’s critical role in disrupting radiative heat transfer pathways [49].

(1) $$\beta =-\frac{\ln\tau }{l\rho }$$

(2) $$L=\frac{Wm_{f}}{A_{l}\rho }$$

where *τ* denotes the infrared transmittance of the nanofiber aerogel, *L* corresponds to the thickness of the compressed KBr pellet, *W* represents the total mass of the KBr mixture, *m_f_* quantifies the mass fraction of aerogel powder within the KBr matrix, and *A_l_* defines the cross-sectional area of the pellet.

**Figure 14:**
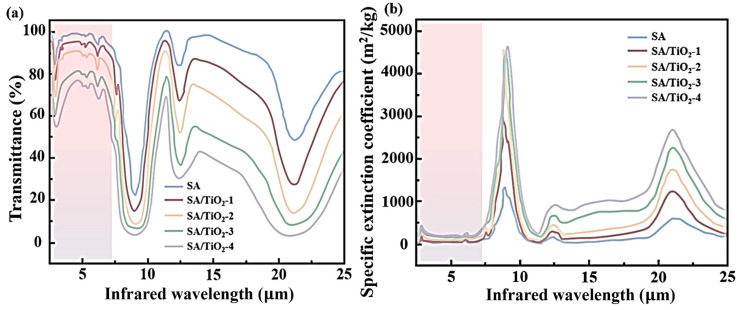
(**a**) Infrared transmittance of nanofibrous aerogels; (**b**) specific extinction coefficient of nanofibrous aerogels.

**Figure 15:**
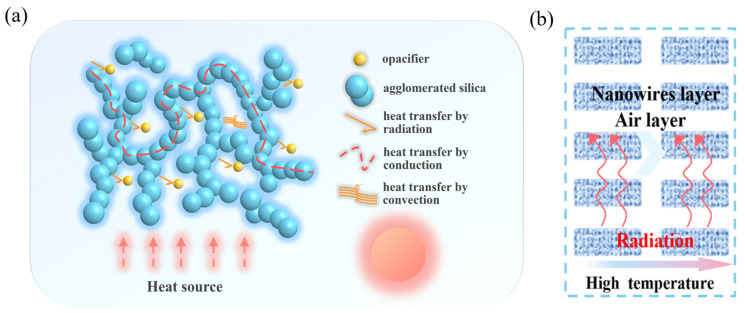
(**a**) Thermal insulation mechanism of SA/TiO_2_ aerogels; (**b**) schematic illustration of heat transfer pathways.

As shown in Figure 14, Fourier-transform infrared (FTIR) spectra were acquired for four groups of SA/TiO_2_ aerogel composites with TiO_2_ doping concentrations ranging from 2.5% to 10%. The results demonstrate a clear correlation between TiO_2_ content and optical performance: as the TiO_2_ doping level increases, the infrared transmittance of the composites decreases significantly, while the mass-specific extinction coefficient exhibits a progressive enhancement. Notably, at the maximum doping concentration of 10%, the mass-specific extinction coefficient reaches 282 m^2^/kg, representing a threefold increase compared to pure SA aerogel. Concurrently, the 10% TiO_2_-doped composite shows the lowest infrared transmittance among all tested samples. These findings directly corroborate the combustion suppression mechanism mediated by TiO_2_ opacifiers (Figure 15a). Specifically, TiO_2_ nanoparticles effectively disrupt radiative heat transfer pathways at elevated temperatures through strong photon attenuation in the near-infrared region. This phenomenon arises from TiO_2_’s higher complex refractive index relative to SiO_2_, which accelerates photon energy dissipation during propagation within the TiO_2_ medium, as supported by prior computational studies [50]. Figure 15b further illustrates the modified heat transfer pathways in SA/TiO_2_ aerogel composites. Quantitative comparisons of thermal insulation efficiency and radiation suppression capabilities across samples with varying TiO_2_ nanoparticle contents are systematically summarized in Table 2.

## 3. Conclusions

This study successfully developed titanium dioxide-doped hydrophobic silica aerogel (SA/TiO_2_) composites and systematically investigated their microstructural characteristics, physicochemical properties, and thermal safety profiles. The optimized SA/TiO_2_ aerogels demonstrated exceptional thermal insulation performance with low density (≤0.12 g/cm^3^), ultralow thermal conductivity (≤18 mW/(m·K)), and significantly reduced combustion heat values. Addressing the inherent limitation of high-temperature infrared transparency in pure silica aerogels, we strategically utilized TiO_2_’s radiation-blocking capability to suppress infrared-mediated heat transfer, achieving dual improvements in thermal safety and insulation efficiency. Remarkably, at TiO_2_ loadings below 10 wt%, the composites exhibited a 55% reduction in combustion calorific value (2.75 MJ/kg), meeting Class A non-combustible material standards, while maintaining thermal conductivity below 18 mW/(m·K). Microscale combustion calorimetry (MCC) revealed TiO_2_’s pronounced suppression effect on combustion intensity, with combined FTIR spectroscopy and post-combustion XRD analysis identifying TiO_2_’s infrared-shielding mechanism as the primary inhibition pathway. Crucially, the opacifier incorporation preserved the aerogel’s nanoporous architecture, as confirmed by BET/BJH analysis showing the maintained surface area and mesopore distribution. This synergistic approach resolves the longstanding trade-off between fire safety and thermal insulation in aerogel materials, enabling high-temperature applications while maintaining structural integrity. The demonstrated compatibility between safety enhancement and performance preservation establishes TiO_2_ doping as a viable strategy for advancing silica aerogels in next-generation thermal management systems.

## 4. Materials and Methods

### 4.1. Materials

The glass fiber reinforcement materials, with diameters ranging from 8 to 15 μm, were obtained from Sinopharm Chemical Reagent Co., Ltd. (SCRC, Shanghai, China). The aerogel composite precursors consisted of sodium silicate solution (34 wt%, modulus = 3.3; Qingdao Dongyue Sodium Silicate Co., Ltd., Qingdao, China) and tetraethyl orthosilicate (TEOS, SCRC). Chemical reagents including absolute ethanol (EtOH), hexamethyl disiloxane (HMDSO), hydrochloric acid (36–38% HCl), sulfuric acid (70%), p-toluenesulfonic acid, and ammonia solution (25–28%) were all of the analytical grades from SCRC. Deionized water (18.2 MΩ·cm) was prepared using an ultra-pure water purification system (ECO-S, HHitech, Shanghai, China). Titanium dioxide nanoparticles (TiO_2_, 10–100 nm particle size) with a diamond-type crystal structure were procured from SCRC.

### 4.2. Preparation of SA/TiO_2_ Aerogel Powders

The SA/TiO_2_ aerogel composite powder was synthesized using TEOS as the silicon source through an ambient pressure drying process. The synthesis began by mixing TEOS, ethanol, 0.1 M hydrochloric acid, deionized water (18.2 MΩ·cm resistivity), and TiO_2_ nanoparticles (10–100 nm diameter) to form a precursor solution. This mixture underwent 5 min of hydrolysis under acidic conditions followed by 30 min of ultrasonication at 40 W output power to ensure uniform dispersion of TiO_2_ nanoparticles within the resulting SA/TiO_2_ sol. Gelation was subsequently induced by adding 0.5 M ammonia solution, achieving a complete phase transition within 10 min. The formed gel was aged in ethanol for 4 h to enhance structural integrity, followed by hydrophobic modification through immersion in HMDSO solution. The final ambient-pressure-dried composite exhibited optimized physical properties, including low density, reduced thermal conductivity, and minimal heat release characteristics, making it suitable for thermal insulation applications. Figure 1a schematically illustrates this TiO_2_ nanoparticle-doped silica aerogel preparation process.

### 4.3. Preparation of SA/TiO_2_ Aerogel Composites

The preparation protocol for aerogel composites, as illustrated in Figure 1b, builds upon the SA/TiO_2_ sol-synthesis methodology outlined in Section 2.1 through strategic incorporation of TiO_2_ nanoparticles during the sol–gel phase, achieving homogeneous dispersion within the silicon precursor matrix before fiber matrix integration. Following composite formation, a 12 h HMDSO-mediated solvent exchange facilitates simultaneous surface modification, with subsequent p-toluene sulfonic acid-catalyzed hydrophobic treatment in HMDSO vapor, optimizing interfacial properties. This engineered process yields SA/TiO_2_ aerogel composites demonstrating enhanced thermal performance characterized by reduced gross calorific value and low thermal conductivity. Comparative analysis of silicon precursors reveals systematic GCV variation: SA/TiO_2_(A) specimens derived from TEOS exhibit lower calorific values than SA/TiO_2_(B) counterparts synthesized via water glass, confirming the universal efficacy of TiO_2_ opacifier integration while highlighting precursor-dependent performance optimization. This dual-source experimental design quantitatively validates the material system’s adaptability across different industrial production frameworks.

### 4.4. Methods for Characterization

Microstructural characterization was performed using field-emission scanning electron microscopy (SEM, SU8220; Hitachi High-Technologies, Tokyo, Japan), while porous architecture analysis was conducted through N_2_ physisorption measurements at 77.10 K employing a 3Flex surface characterization system (Micromeritics, Norcross, GA, USA). The Brunauer–Emmett–Teller (BET) method was applied to determine specific surface area, with pore size distribution and total pore volume derived from Barrett–Joyner–Halenda (BJH) analysis of adsorption isotherms. Hydrophobicity assessment involved contact angle measurements using an SL200K goniometer (KINO Industry Co., New York, NY, USA). Chemical functionality was probed through Fourier-transform infrared spectroscopy (FT-IR, Nicolet iS20, Thermo Fisher Scientific, Waltham, MA, USA), with spectral acquisition performed in transmission mode across the 4000–400 cm^−1^ range at 4 cm^−1^ resolution. The bulk density (*ρ*_b_) was approximated by a tap density, which was measured using a tap density meter (ZS-202, Liaoning Instrument Research Institute Co., Ltd., Dandong, China) with 300 r/min for continuous vibration within 10 min. The porosity is calculated according to the following formula:

(3) $$Porosity=\left(1-\frac{\rho_{b}}{\left(1-c\right)\rho_{\left(s-SiO_{2}\right)}+c\rho_{s-dopat}}\right)\times 100\%$$

where *c* is the additive content of hydroxides, *ρ*_*s−SiO*2_ is the skeletal density, usually 2.2 g/cm^3^, and −*ρ_s−dopat_* is the skeletal density of the dopants.

The thermal conductivity of the samples was measured using a laser flash analyzer (LFA 467, NETZSCH Group, Selb, Germany) combined with a heat flow meter ((HFM 446S, NETZSCH Group, Selb, Germany). Thermogravimetric analysis (TGA) was conducted on a NETZSCH TG 209 F3 instrument (NETZSCH Group, Selb, Germany) under nitrogen flow (30 mL min^−1^) with samples heated from 50 to 900 °C at a constant heating rate of 10 °C min^−1^. Microscale combustion calorimetry (MCC) was performed using an FAA-PCFC system (Thoron GmbH, Bochum, Germany), where specimens underwent programmed heating from 50 to 700 °C at 1 °C s^−1^ under 80 mL min^−1^ nitrogen flow, followed by isothermal combustion at 700 °C in a 20/80 (*v*/*v*) O_2_/N_2_ mixture (total flow rate 100 mL min^−1^). Gross calorific value (GCV) determination was carried out with an IKA C3000 oxygen bomb calorimeter (IKA Werke GmbH & Co. KG, Staufen, Germany) following ISO 1716:2002 specifications [51]. Post-combustion residues were characterized by X-ray diffraction (Empyrean XRD, Malvern Panalytical B.V., Almelo, The Netherlands) employing Cu-Kα radiation (λ = 1.5406 Å). This comprehensive analytical protocol systematically evaluates the thermal transport properties, decomposition kinetics, combustion characteristics, energy content, and phase composition of residues.

## Figures and Tables

**Table 1:** Physical properties of pure SA, SA/TiO_2_ composites with varying TiO_2_ loadings, and pure TiO_2_ nanoparticles.

Samples	BET Surface Area (m^2^/g)	Pore Volume (cm^3^/g)	Average Pore Size (nm)
Pure SA	682.89	0.11	9.38
5% TiO_2_/SA	658.28	0.14	10.82
10% TiO_2_/SA	370.57	0.14	13.22
15% TiO_2_/SA	361.59	0.24	13.29
20% TiO_2_/SA	275.46	0.25	13.34
TiO_2_	5	0.03	42.44

**Table 2:** Effects of TiO_2_ nanoparticle content on thermal insulation and radiative suppression properties in nanofibrous aerogels.

Concentration of TiO_2_ (%)	Thermal Conductivity (W/mK)	Infrared Transmittance (%, at 3 μm)	Specific Extinction Coefficient (m^2^/kg, at 3 μm)
2.5	0.017	92	98
5	0.019	88	142
7.5	0.018	76	189
10	0.018	68	282

## Data Availability

The data presented in this study are available on request from the corresponding author.
